# Obese visceral adipose dendritic cells downregulate regulatory T cell development through IL-33

**DOI:** 10.3389/fimmu.2024.1335651

**Published:** 2024-03-19

**Authors:** Shindy Soedono, Sharlene Sharlene, Dan Hoang Nguyet Vo, Maria Averia, Eufrasia Elaine Rosalie, Yun Kyung Lee, Kae Won Cho

**Affiliations:** ^1^ Department of Integrated Biomedical Science, Soonchunhyang University, Cheonan, Republic of Korea; ^2^ Soonchunhyang Institute of Medi-bio Science (SIMS), Soonchunhyang University, Cheonan, Republic of Korea; ^3^ Magister of Biotechnology, Atma Jaya Catholic University of Indonesia, Jakarta, Indonesia; ^4^ Faculty of Biotechnology, Department of Food Technology, Atma Jaya Catholic University of Indonesia, Jakarta, Indonesia

**Keywords:** regulatory T cell, obesity, inflammation, adipose tissue, visceral adipose tissue, dendritic cell, IL-33, macrophage

## Abstract

Regulatory T cells (Tregs) residing in visceral adipose tissue (VAT) play a pivotal role in regulating tissue inflammation and metabolic dysfunction associated with obesity. However, the specific phenotypic and functional characteristics of Tregs in obese VAT, as well as the regulatory mechanisms shaping them, remain elusive. This study demonstrates that obesity selectively reduces Tregs in VAT, characterized by restrained proliferation, heightened PD-1 expression, and diminished ST2 expression. Additionally, obese VAT displays distinctive maturation of dendritic cells (DCs), marked by elevated expressions of MHC-II, CD86, and PD-L1, which are inversely correlated with VAT Tregs. In an *in vitro* co-culture experiment, only obese VAT DCs, not macrophages or DCs from subcutaneous adipose tissue (SAT) and spleen, result in decreased Treg differentiation and proliferation. Furthermore, Tregs differentiated by obese VAT DCs exhibit distinct characteristics resembling those of Tregs in obese VAT, such as reduced ST2 and IL-10 expression. Mechanistically, obesity lowers IL-33 production in VAT DCs, contributing to the diminished Treg differentiation. These findings collectively underscore the critical role of VAT DCs in modulating Treg generation and shaping Treg phenotype and function during obesity, potentially contributing to the regulation of VAT Treg populations.

## 1 Introduction

Regulatory T cells (Tregs) are a specialized subset of CD4^+^ T cells that play pivotal roles in maintaining immune tolerance and suppressing immune response in both health and disease (1). Recently, there has been a heightened interest in adipose tissue (AT) Tregs, primarily due to their significant association with metabolic dysfunctions in diseases and aging (2–10). Notably, visceral epididymal AT (VAT) harbors a uniquely high number of Foxp3^+^ Tregs, which are reduced in obesity (5, 7, 9, 11, 12). Conversely, aged mice show a greater accumulation of VAT Tregs (10). These observations indicate the presence of distinct regulatory mechanisms governing VAT Tregs, which may vary under different conditions and affect their functionality. Particularly in response to obesity, the decreased number of Tregs has been implicated in contributing to the progression of chronic inflammation within VAT (5, 9, 13, 14). However, there remains a gap in understanding fat depot specific Treg regulation, including their phenotypic and functional characteristics, as well as the mechanisms governing their features and function during obesity.

The presence of Tregs in tissues can originate from lymphoid organs or be induced peripherally through interactions with antigen presenting cells (APCs) (12, 15–17). The distinct locations of VAT and subcutaneous inguinal AT (SAT) have been suggested to influence the origin and development of Tregs (18). In murine studies, it has been reported that Tregs primarily derived from thymocytes seed into VAT early in life. As mice mature, VAT Treg populations expand up to 80% of CD4^+^ T cells, while SAT consistently constitutes about 10% of CD4^+^ T cells (19). Additionally, Tregs in VAT exhibit unique and diverse T cell receptor (TCR) sequences with specific clonality dependencies for their accumulation (5, 19, 20). These findings suggest the presence of local regulators that shape, nurture, and modulate the distinct Treg phenotype and features in different fat depots. Notably, APCs have been implicated as potential local factors in orchestrating Tregs within VAT (19, 21).

Communication with local APCs is a prominent mechanism involved in regulating the proliferation, survival, and activation of Tregs. The accumulation of Tregs in VAT has been indicated to depend on antigens presented on MHC-II molecules by APCs. Deletion of MHC-II molecules or treatment with anti-MHC-II antibodies resulted in a diminished Treg population in VAT (19, 20, 22). Furthermore, co-stimulatory molecules CD80/CD86 has been reported to be required for maintaining Treg number in VAT (23, 24). Additionally, the interaction of PD-L1 on APCs with PD-1 on T cells has been identified as the essential pathway for inducing Foxp3 expression during Treg differentiation by inhibiting PI3K/AKT/mTOR pathway (25). However, AT contains various types of APCs, such as macrophages (Mφs) and dendritic cells (DCs), which require careful separation using CD64 or MerTK due to overlapping CD11c expression in obesity conditions (26, 27). Therefore, it remains unclear whether obesity alters the expressions and functions of their antigen-presenting molecules. Importantly, their individual capability to regulate the Treg population in obesity remains unknown.

In addition to direct interaction, cytokines signaling also plays a pivotal role in regulating Treg development (28). Aside from the indispensable cytokines such as IL-2 and TGFβ, interleukin-33 (IL-33) has been identified as a critical driver for Treg accumulation with controversial mechanisms of action, either via intermediary cells (29–32) or by directly affecting Tregs (19–21). Notably, high expression levels of ST2, the IL-33 receptor, were observed in VAT Tregs (21, 33). Moreover, both gain and loss-of-function models involving IL-33 or ST2 have demonstrated specific alterations to the Treg populations in VAT, indicating IL-33/ST2 axis as a crucial mechanism to control Treg populations during obesity (21, 34–36). While diverse cell types express and release IL-33 in VAT (34, 36–40), recent evidence has indicated that DCs also express IL-33, potentially contributing to Treg regulation (41). However, it remains uncertain whether IL-33 is indeed expressed in adipose tissue dendritic cells (ATDCs) and whether it can mediate Treg regulation, particularly in the context of obesity.

Using the *in vitro* co-culture studies involving APCs and T cells, various research groups, including our own, have previously demonstrated the capacity of both ATDCs and AT macrophages (ATMs) to induce T cell differentiation, with ATDCs exhibiting the most potent effect (26, 42). However, the relative contribution of ATDCs and ATMs in regulating Treg in obese conditions and the underlying mechanisms has remained unclear. In this study, our objective is to investigate whether Tregs in VAT and SAT are differentially regulated by specific types of APCs in the context of obesity. Our observations revealed that obesity led to a distinct Treg profile in VAT, characterized by restrained proliferation, higher PD-1 and lower ST2 expressions. In addition, obese VAT displayed a higher number of ATDCs marked by elevated MHC-II, CD86, and PD-L1 expressions, which inversely correlated with AT Tregs. Furthermore, using an *in vitro* co-culture system, we found that obese VAT DCs were the sole APCs to decrease Treg differentiation, exhibiting lower proliferation, reduced ST2 and IL-10 expressions, as well as increased phospho-AKT/-mTOR and PD-1 expressions. Moreover, this study discovered that obesity diminished the expression and secretion of IL-33 derived from ATDCs, which is essential for driving Treg differentiation. Overall, our studies uncover the potential significant contributions of ATDCs in orchestrating Treg population in visceral fat during obesity.

## 2 Materials and methods

### 2.1 Animals and metabolic evaluations

C57BL/6J and OT-II (B6.Cg-Tg(*TcraTcrb*)425Cbn/J) mice were obtained from Jackson Laboratories and maintained in specific pathogen-free animal facility with a 12 h dark/light cycle. Six weeks old male C57BL/6J mice were assigned to normal diet (ND; PicoLab^®^ Mouse Diet 20 #5058) or high fat diet (HFD; Research Diets Inc. #D12492) for 16 weeks and the body weights were recorded weekly. At 12 weeks of HFD, fasting blood glucose was measured with a glucometer (Barozen, Korea) after 16 h of fasting. All mice procedures were approved by the Institutional Animal Care and Use Committee at the Soonchunhyang University (SCH20-0014).

### 2.2 Cells isolation and flow cytometry

The stromal vascular cells (SVCs) were isolated from ATs and splenocytes were isolated from spleen as previously described (43). In brief, tissues were dissected and mechanically minced for digestion with 2 mg/mL collagenase II (Sigma Aldrich) for SVCs and 1 mg/mL collagenase II for splenocytes at 37°C for 30 min with shaking. Digested cells were then filtered and centrifuged at 500xg 4°C for 5 min. Subsequently, cells were undergoing RBC lysis for 5 min at RT. Cells were then counted and incubated with CD16/CD32 antibody for 10 min at 4°C and stained with indicated antibodies for 30 min at 4°C. For Tregs, cells were fixed and permeabilized using Foxp3/Transcription Factor Staining Buffer Set (eBiosciences), then further stained with Anti-Mouse Foxp3 antibody for 2 h at 4°C. Flow cytometry analyses were performed on BD FACS Canto™ II (Beckon Dickinson) and analysed with FlowJo software (Treestar). All antibodies were listed in **Supplementary Table 1**.

### 2.3 Cell sorting of antigen presenting cells and naïve CD4^+^ T cells

Cell sorting was performed using BD FACS Aria Cell Sorting System. For ATDCs and ATMs from VAT and SAT, isolated SVCs were stained for Live/Dead-e506, CD45-e450, CD64-PE, and CD11c-APC. Live CD45^+^ cells were then gated for ATDCs (CD64^-^ CD11c^+^) and ATMs (CD64^+^). For splenic DC, isolated splenocytes were enriched for CD11c^+^ cells by staining with CD11c-APC and anti-APC microbeads (Miltenyi Biotech Inc). Isolated cells were further stained with Live/Dead-e506, CD45-e450, CD3-PE, and CD11c-APC, then sorted using gating Live CD45^+^ CD3^-^ CD11c^high^. For naïve CD4^+^ T cells, isolated splenocytes were negatively sorted using MACS Naïve CD4^+^ T cell Isolation Kit following manufacturers protocol (Miltenyi Biotech Inc).

### 2.4 CFSE labelling

For antigen-specific assay, naïve CD4^+^ T cells were isolated from spleen of OT-II mice. Isolated cells were pelleted and resuspended in 1 mL PBS. After warming at 37°C 5 min, Cell Trace CFSE (Invitrogen) was added into cells (2 µM) and incubated at 37°C 5 min. The labelling reaction was stopped by adding 10 mL of cold media and further incubated on ice for 10 min.

### 2.5 *In vitro* co-culture for Treg differentiation

Isolated APCs and naïve CD4^+^ T cells were seeded in 1:5 ratio for five days co-culture using 96 well U bottom in RPMI cold complete media supplemented with 10% FBS (R&D), 1% Penicillin-streptomycin, 1% MEM, 1% sodium pyruvate, and 0.1% 2-mercaptoethanol. Treg differentiation was induced by adding 1 µg/mL anti-CD3, 20 ng/mL recombinant murine IL-2, and 5 ng/mL recombinant human TGF-β. In antigen-specific assay, sorted APCs were seeded and treated with 20 ng/mL OVA_323-339_ for 3 h at 37°C prior to addition of CFSE-labelled naïve CD4^+^ T cells. In blocking experiments, sorted APCs were seeded and treated with anti (α)-IL-33 (1 µg/mL), α-PD-L1 (5 µg/mL), or α-CD86 (5 µg/mL) for 2 h at 37°C prior to addition of naïve CD4^+^ T cells. For treatment with exogenous IL-33, co-culture was treated with 20 ng/mL recombinant murine IL-33 (rIL-33).

At day 5 of Treg differentiation, cells were harvested and stained for surface markers Live, CD4, ST2, and PD-1. Subsequently, cells were permeabilized and stained with intracellular antibody Foxp3 as described before. To detect intracellular cytokines expression, cells were activated with PMA/Ionomycin/Golgi plug mix for 5 h at 37°C prior to the surface staining and intracellular staining. In the antigen-specific assay, median fluorescence intensity (MFI) of CFSE was determined with FlowJo and the proliferation score (PS) was calculated following previous study (44), defined as the median of Treg division at day 5 relative to non-stimulated T cells.

### 2.6 Bone marrow-derived dendritic cells differentiation and treatment

Bone marrow (BM) cells were isolated from femur and tibia by flushing. BM cells were cultured in complete DMEM (10% FBS) and supplemented with 20 ng/mL of recombinant murine GM-CSF (Peprotech). On day 3, fresh media containing GM-CSF was added to the culture. On day 6 and 8, half of the media was collected, centrifuged, and the pellet was resuspended with fresh media containing GM-CSF then given back to the culture. On day 10, mature BMDCs were harvested and seeded for further treatment.

To prepare adipose tissue conditioned media (ATCM), VAT was isolated and incised into around 1 mm^3^. Tissues were then cultured in DMEM containing 10% FBS for 24 h. On the next day, the tissues were washed with PBS and cultured in serum starvation media containing 0.1% FBS for another 24 h. Media was then collected, centrifuged, and filtered through 0.2 µm before stored at -80°C. For cell treatment, 25% of ATCM from ND and HFD VAT was added to the mature BMDC for 24 h. Separately, vehicles (10% BSA) or palmitate-BSA (0.5 mM/L) were added to BMDCs for 24 h. Media were collected for determination of IL-33 levels.

### 2.7 ELISA of IL-33

IL-33 levels were measured using Mouse IL-33 Duo Set ELISA (R&D systems, #DY3626) following manufacturers protocol. For determination of IL-33 levels by VAT DCs, 2x10^4^ sorted cells from lean and obese mice were seeded in 96 wells for 24 h and media were collected.

### 2.8 Gene expression analysis

Total RNA was extracted from cells using RiboEx (Geneall). The RNA was quantified and reversed transcribed into cDNA by using High-Capacity cDNA Reverse Transcription Kit (Applied Biosystem). SYBR Green PCR master mix was used, and quantitative real-time PCR reaction was performed using Quant Studio 1. Levels of mRNA expressions were calculated using the 2^−ΔΔCT^ method relative to the expression level of 18S ribosomal RNA. Following primers were used in the reaction: *Il33* F: TCCAACTCCAAGATTTCCCCG; *Il33* R: CATGCAGTAGACATGGCAGAA; *18s* F: TTGACGGAAGGGCACCACCAG; *18s* R: GCACCACCACCCACGGAATCG.

### 2.9 Immunostaining

Adipose tissues were fixed in 1% PFA overnight at 4°C. For immunohistology, fixed tissues were proceeded into paraffin tissue sections, then subsequently stained with Hematoxylin and Eosin (H&E) staining. For whole-mount tissue staining, fixed tissues were incised into around 0.5 cm^3^. Tissues were stained with Caveolin and CD4 overnight at 4°C, and followed by secondary antibodies for 2 h at RT. For immunofluorescence (IF) staining in sorted VAT DC, 100 µL suspension of sorted cells (~20,000 cells) were attached to glass slides with Cytospin at 2000 rpm 7 min. Slides were then fixed with 4% PFA for 10 min at RT, washed in PBS, and dried overnight at RT. After cell permeabilization, slides were incubated with IL-33 antibody (R&D system) overnight at 4°C. Images were taken with Leica DMi8 or Confocal microscopy (Zeiss LSM 710).

### 2.10 Statistical analysis

All values reported as mean ± standard error mean (SEM), or otherwise stated. Statistical differences were determined using the unpaired Student t test, one-way ANOVA, or two-way ANOVA for multiple groups with Tukey’s multiple comparisons test using GraphPad Prism V8.0. All data were normalized to ND or control group. Statistical significance represented as *p < 0.05, **p < 0.01, ***p < 0.001, ****p < 0.0001 unless otherwise indicated.

## 3 Results

### 3.1 Obese visceral adipose tissue has less regulatory T cells with distinct phenotypes.

To explore the depot-specific profile of AT Tregs in obesity, male mice were fed either a normal chow diet (ND) or a high-fat diet (HFD) for 16 weeks. As expected, HFD-fed mice exhibited increased body weight, higher adiposity, and hyperglycemia compared to ND-fed mice, confirming the success of the diet-induced obesity model (**Supplementary Figure 1A**). Histological analysis of adipose tissues revealed an increase immune cells infiltration with crown like structures (CLS) only in VAT but not in SAT (**Supplementary Figure 1B**).

**Figure 1:**
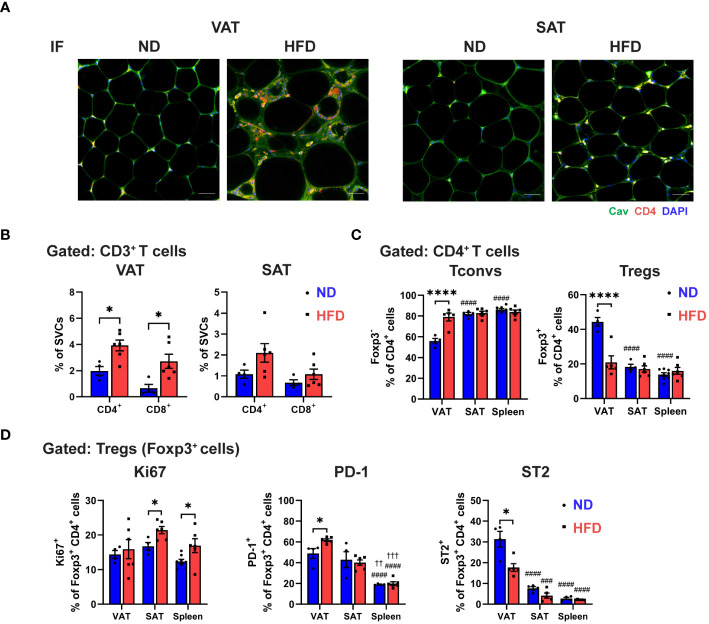
Distinctive regulatory T cell (Treg) phenotype in obese visceral adipose tissue (VAT). C57BL/6 mice were induced to diet-induced obesity (DIO) by 16 weeks of high-fat diet (HFD). **(A)** Immunofluorescence (IF) staining of Caveolin (green) and CD4 (red) in visceral adipose tissue (VAT) and subcutaneous AT (SAT) (scale bar=50 µm). **(B-D)** Flow cytometry analysis of stromal vascular cells (SVCs) isolated from VAT and SAT, and splenocytes isolated from spleen. **(B)** Quantification of CD4^+^ and CD8^+^ T cells in VAT and SAT in percentage of SVCs. **(C)** Quantification of Foxp3^-^ conventional T cells (Tconvs) and Foxp3^+^ regulatory T cells (Tregs) in percentage of CD4^+^ cells. **(D)** Expressions of Ki67, PD-1, and ST2 in Tregs were determined and quantified. Data are means ± SEM, n= 4-6, *p<0.05, ****p<0.0001 ND vs HFD. ###*p*<0.001, ####*p*<0.0001 VAT vs SAT or spleen. ††*p*<0.01,††† *p*<0.001 SAT vs spleen. ND, normal diet; HFD, high-fat diet.

Next, we assessed the CD4^+^ T cells population in obese ATs by using immunofluorescence (IF) staining. The results showed an elevated amount of CD4^+^ T cells in obese VAT, but not in SAT (**Figure 1A**). Furthermore, we utilized flow cytometry to assess T cell profile (**Supplementary Figure 1C**), and consistently, the frequency of CD4^+^ T cells, as well as CD8^+^ T cells, from total SVCs were significantly increased only in obese VAT, but not in SAT (**Figure 1B**). Within the CD4^+^ T cells population, conventional T cells (Tconv; Foxp3^-^) were induced in obese VAT, while Tregs were markedly decreased from approximately 40% to about 20%. No alterations were observed in Tconv and Treg populations in obese SAT and spleen (**Figure 1C**). Additionally, Treg numbers were reduced by 3.5-fold in obese VAT but not in SAT (**Supplementary Figure 1D**), indicating depot-specific adipose Treg regulation in obesity.

To further explore obesity-specific phenotypic characteristic of Tregs in distinct fat depots, we examined the Treg proliferation and exhaustion by analyzing the expressions of Ki67, PD-1, and ST2 (**Figure 1D**; **Supplementary Figure 1E**). In lean state, the frequency of proliferating Tregs (Ki67^+^ Tregs) in VAT, SAT, and spleen was comparable. However, HFD feeding significantly increased the frequency of Ki67^+^ Tregs in SAT and spleen, but not in VAT, suggesting that VAT Treg depletion could be attributed to differential regulation of adipose Treg proliferation in obesity. Furthermore, a similar frequency of PD-1^+^ expressing Tregs was observed in lean VAT and SAT, which was higher compared to the spleen. However, a significant increase in PD-1^+^ expressing Tregs was found only in obese VAT, not in SAT and spleen. Yet, quantification of mean fluorescence intensity (MFI) showed no difference in PD-1 expression in obese Tregs from all tissues. Additionally, our data validated ST2 as a signature marker of VAT Tregs in the steady state, where around 30% of Foxp3^+^ Tregs expressed ST2, while SAT and spleen displayed less than 10%. The reduced number of ST2^+^ expressing Tregs was detected in obese VAT, contributing to the lower cell number of ST2^+^ Tregs, but not in SAT and spleen. Overall, these results indicate that obesity leads to a specific reduction of Tregs in VAT, marked by restrained proliferation, elevated PD-1 expressions, and reduced ST2 levels, signifying the uniqueness of Treg regulation in VAT.

### 3.2 Obesity leads to depot-specific differential regulation of macrophages and dendritic cells in adipose tissue.

**Figure 2:**
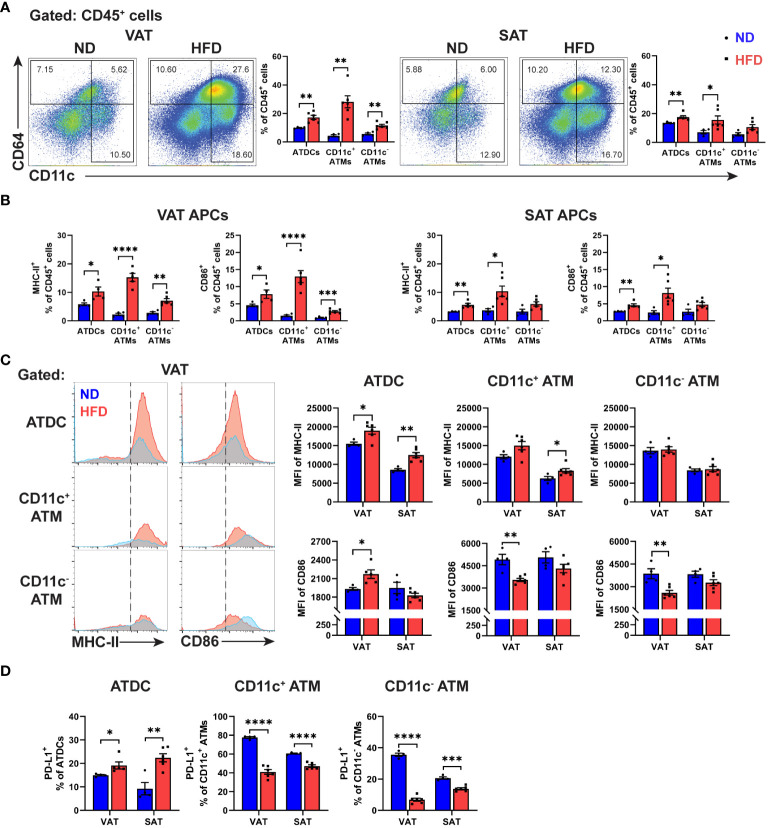
Differential expressions of antigen presentation molecules in macrophages and dendritic cells from obese VAT and SAT. **(A)** Representative flow cytometry and quantification of ATDC (CD64^-^ CD11c^+^), CD11c^+^ ATM (CD64^+^ CD11c^+^), and CD11c^-^ ATM (CD64^+^ CD11c^-^) in percentage of leukocytes (CD45^+^). **(B)** Quantitation of MHC-II^+^ and CD86^+^ in VAT and SAT APCs in percentage of leukocytes. **(C)** Representative histogram and quantification of median fluorescence intensity (MFI) of MHC-II and CD86 in VAT and SAT APCs. **(D)** Quantification of PD-L1^+^ cells in APCs in VAT and SAT. Data are means ± SEM, n= 4-6, *p<0.05, **p<0.01, ***p<0.001, ****p<0.0001 ND vs HFD. ND, normal diet; HFD, high-fat diet.

We next analysed the antigen presenting cells (APCs) phenotype in ATs of lean and obese mice, using CD64 to carefully to distinguish between ATDCs (CD64^-^) and ATMs (CD64^+^) (26). Obesity increased the frequency of all APC types, including ATDCs, CD11c^+^ ATMs, and CD11c^-^ ATMs, in both VAT and SAT, with a more pronounced effect in VAT (**Figure 2A**; **Supplementary Figure 2A**). Additionally, obesity induced the expression of MHC-II and CD86 in all APCs in both VAT and SAT (**Figure 2B**; **Supplementary Figure 2B**). However, in VAT, the mean fluorescence intensity (MFI) of MHC-II was only increased in ATDCs, while SAT exhibited elevated expressions in ATDCs and CD11c^+^ ATMs. Notably, a significant increase in the MFI of CD86 was observed only in VAT ATDCs, not in SAT. Interestingly, different APC types displayed distinct expression patterns, with both CD11c^+^ and CD11c^-^ ATMs showing reduced MFI of CD86 expressions in obese VAT but not in SAT (**Figure 2C**). Furthermore, we evaluated the expressions of PD-L1 and found that, in lean state, a high frequency of PD-L1^+^ cells were observed in CD11c^+^ ATMs, followed by CD11c^-^ ATMs then ATDCs in both VAT and SAT. However, in obesity, the proportion of PD-L1^+^ cells increased only in ATDCs, while CD11c^+^ and CD11c^-^ ATMs displayed lower PD-L1^+^ expressions both in VAT and SAT (**Figure 2D**). These results collectively reveal that obesity distinctively alters the expression of molecules associated with antigen presentation and T cell interaction in ATDCs and ATMs within VAT and SAT.

**Figure 3:**
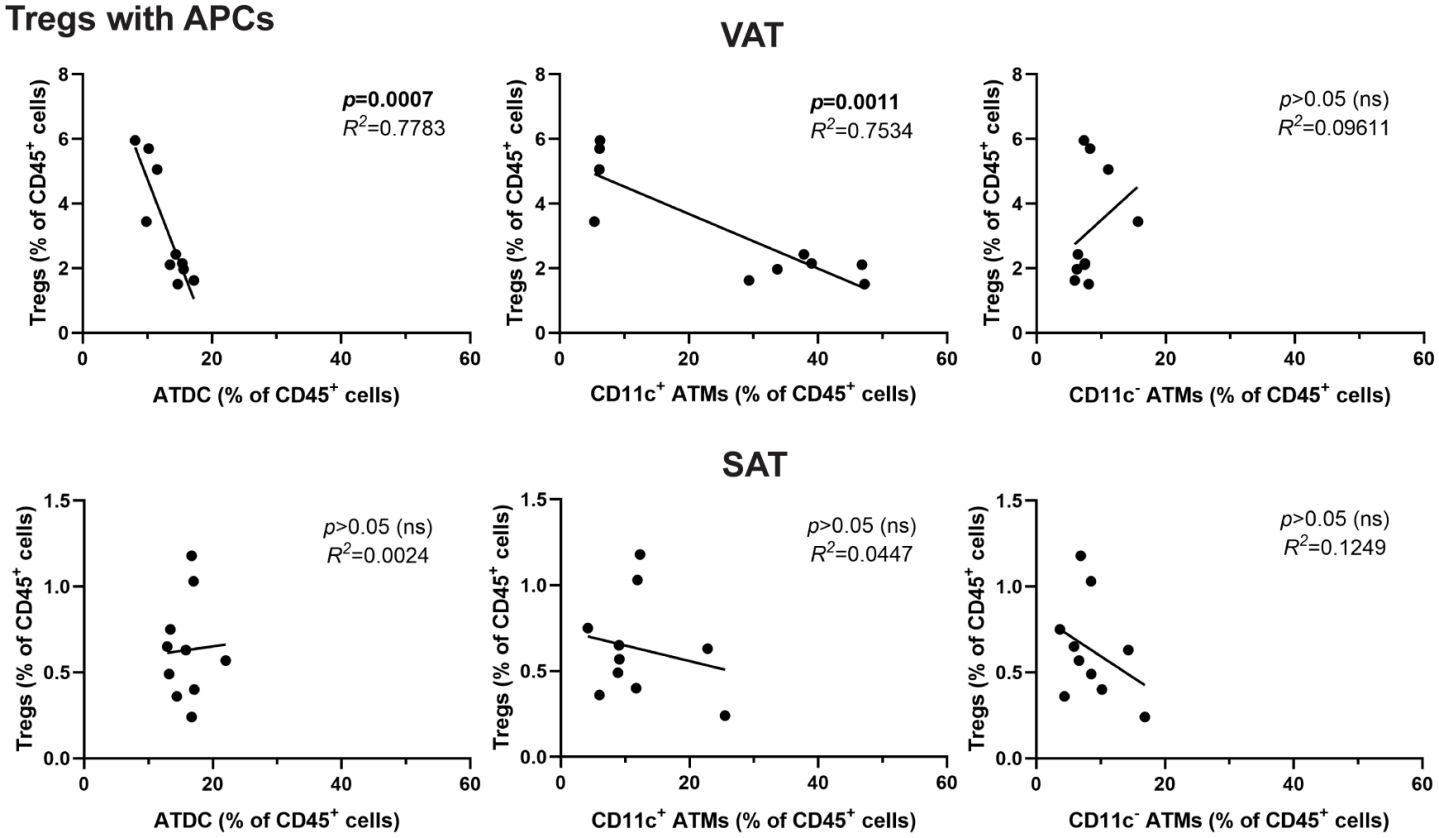
Inverse correlation between Tregs and DCs in obese VAT but not SAT. Correlation analysis of APCs (ATDC, CD11c^+^ ATM, and CD11c^-^ ATM) with Tregs in percentage of leukocytes. Data are means ± SEM, n= 4-6, ND, normal diet; HFD, high-fat diet; ns, not significant.

Given the distinct characteristics of Tregs and different APC types in obesity, we further explored their potential relationship by conducting correlation analyses. In VAT, but not in SAT, the frequency of Tregs was negatively correlated with ATDCs and CD11c^+^ ATMs (**Figure 3**). Moreover, we attempted to analyse between Tregs and CD86-expressing cells in APCs. The results showed distinct correlation patterns in VAT, where CD86 expressing ATDCs exhibited a negative correlation, while both CD11c^+^ and CD11c- ATMs showed positive correlations (**Supplementary Figure 3A**). Similar patterns were observed in the analysis of PD-1^+^ Tregs and PD-L1^+^ APCs, with a positive correlation between PD-1^+^ Tregs and PD-L1^+^ ATDC, while both CD11c^+^ and CD11c^-^ ATM displayed negative correlations (**Supplementary Figure 3B**). These results imply that different APC types in VAT may have varying regulation to Treg populations during obesity.

### 3.3 Obese VAT DCs, but not macrophages or in different locations, reduces Treg differentiation in both antigen-specific and non-specific manners.

**Figure 4:**
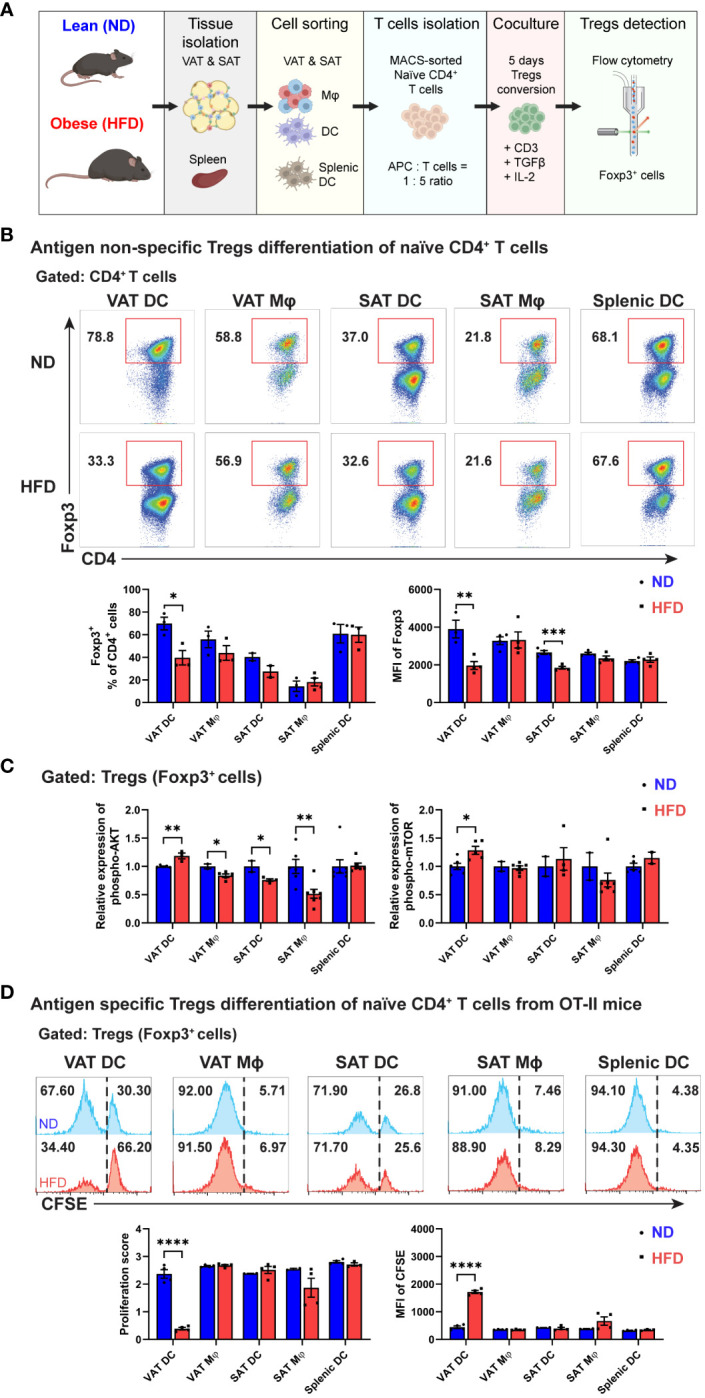
Decreased Treg differentiation by obese VAT DC both in antigen non-specific and antigen-specific manner. **(A)** Schematic diagram of *in vitro* co-culture for Tregs differentiation. **(B)** Representative flow cytometry diagrams and quantification of Foxp3^+^ cells population among CD4^+^ T cells on day 5. MFI of Foxp3 were determined for each Tregs generated by specific APCs. **(C)** Relative expressions of phospho-AKT (Ser473) and phospho-mTOR (Ser2448) in Tregs were determined and quantified after permeabilization and intracellular staining. **(D)** Representative flow cytometry diagrams and quantification of CFSE expression in Foxp3^+^ cells after five days co-culture in antigen-specific manner. Proliferation score and MFI of CFSE in Tregs were determined and quantified. Data are means ± SEM, n= 3-5, *p<0.05, **p<0.01, ***p<0.001 ****p<0.0001 ND vs HFD. ND, normal diet; HFD, high-fat diet.

Distinct correlation patterns between ATDCs and ATMs with Tregs in the VATs from obese mice prompted an investigation into the potential of resident APCs within ATs to regulate Treg populations using the *in vitro* Treg differentiation co-culture system. Following a five-day co-culture with various APC types, including VAT DCs, VAT Mφs, SAT DCs, SAT Mφs, and splenic DCs, the generation of Foxp3^+^ cells was quantified using flow cytometry (**Figure 4A**). While all APC types exhibited the potential to induce Treg differentiation, a fat depot-specific regulation of Treg induction potentials was observed, with VAT DCs and Mφs demonstrating a higher capability compared to their SAT counterparts. Interestingly, among all APCs, obese VAT DCs were the only ones with a diminished capacity to induce Treg differentiation, a phenomenon not observed in other APCs. Alongside a significant reduction in Foxp3^+^ cells, obese VAT DCs exhibited a more pronounced decreases in the MFI of Foxp3 in generated Tregs compared to obese SAT DCs (**Figure 4B**). Subsequently, an examination of whether obese VAT DCs specifically led to decreased Foxp3^+^ cells during Treg differentiation was conducted by analyzing AKT/mTOR signalling in Tregs. Consistent with Foxp3^+^ frequency data, higher expressions of phospho-AKT and -mTOR were identified only in the Tregs co-cultured with obese VAT DC, while VAT Mφs, SAT DCs, and SAT Mφs exhibited reduced expressions of phospho-AKT (**Figure 4C**).

Given the antigen-non-specific design of our *in vitro* co-culture system, we further investigated the regulation of APCs on Treg differentiation in an antigen-specific manner using OVA-specific CD4 T cells from OT-II mice. Remarkably, among various APC types, obese VAT DCs were the exclusive APCs that reduced the proliferation of carboxyfluorescein succinimidyl ester (CFSE)-labeled Foxp3^+^ cells. The higher proportion of undivided CFSE^+^ cells correlated with a lower proliferation score and significantly higher MFI of CFSE in Tregs co-cultured with obese VAT DC (**Figure 4D**). In summary, these findings highlight obese VAT DCs as the primary APCs driving the reduction in Treg generation *in vitro*, potentially modulating the population and numbers of Tregs in VAT.

### 3.4 Obese VAT DC drives unique phenotype and function of Tregs.

**Figure 5:**
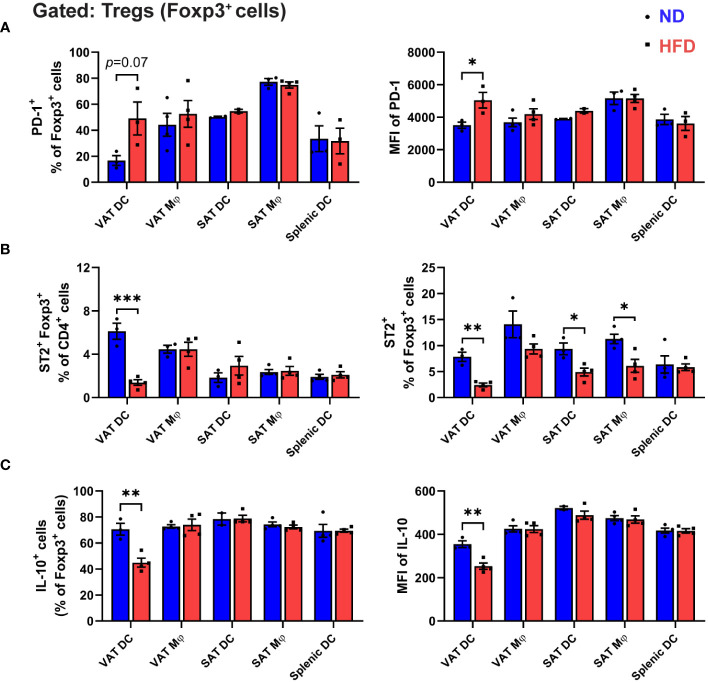
Unique phenotypes of Treg co-cultured with obese VAT DC. **(A-C)** On day 5 of *in vitro* co-culture with specific APCs, Foxp3^+^ cells profiles were analyzed with flow cytometry. **(A)** Quantification of PD-1^+^ cells and MFI of PD-1. **(B)** Frequency of ST2^+^ cells Tregs among CD4^+^ T cells and Foxp3^+^ cells. **(C)** Quantification of intracellular cytokine IL-10 expressions in Foxp3^+^ cells after cells activation and permeabilization. Data are means ± SEM, n= 3-5, *p<0.05, **p<0.01, ***p<0.001 ND vs HFD. ND, normal diet; HFD, high-fat diet.

Subsequently, we assessed the capacity of APCs to shape the specific phenotype of *in vitro*-differentiated Tregs by examining the expressions of PD-1 and ST2. Notably, a marked elevation in PD-1 expression was discerned in Tregs generated by obese VAT DCs, a phenomenon not observed with other APCs (**Figure 5A**). Furthermore, while all APCs demonstrated capabilities in generating ST2^+^ Tregs, VAT DCs, in the context of obesity, exhibited a notably significant decrease in ST2^+^ expression among Tregs. This resulted in a reduced proportion of ST2^+^ Foxp3^+^ cells within the CD4^+^ T cells (**Figure 5B**).

To delve deeper into the impact of obese VAT DCs on the functionality of *in vitro*-differentiated Tregs, we scrutinized the intracellular expressions of signature Treg cytokine interleukin-10 (IL-10). In contrast to the lean condition, a substantial reduction in IL-10-expressing Tregs was evident only when co-cultured with obese VAT DCs, not with other APCs (**Figure 5C**). Furthermore, an inclination towards increased IL-17-A and interferon-gamma (IFN-γ) expression was observed in Tregs generated by obese VAT DCs, indicating a less anti-inflammatory profile (**Supplementary Figure 4**). Collectively, these findings underscore the pivotal role of VAT DCs as the primary regulators directing the attributes and function of *in vitro* differentiated Tregs, resembling the distinctive phenotypes of Tregs in obese VAT.

### 3.5 PD-L1 and CD86 signalling are involved in Treg development, but this involvement is not exclusive to obese VAT DCs.

**Figure 6:**
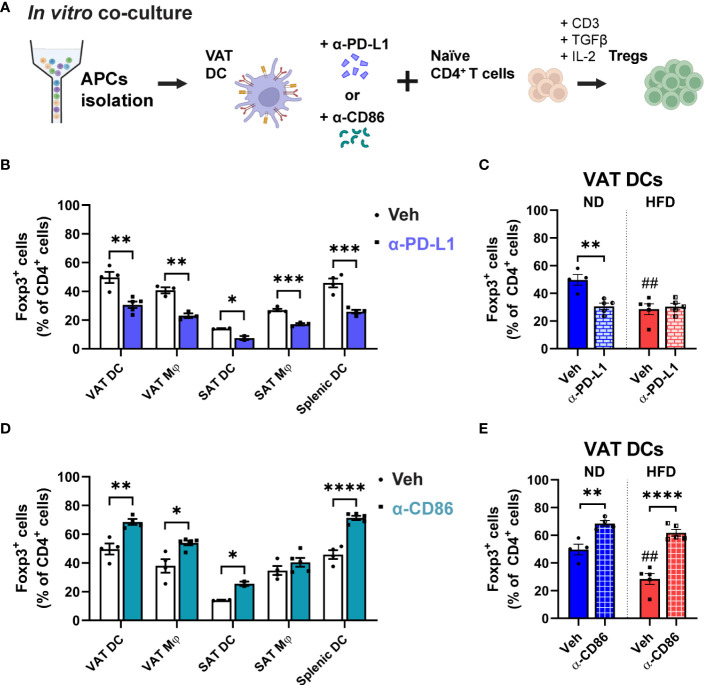
Involvement of PD-L1 and CD86 in VAT DC-Treg interactions. **(A)** Schematic illustration of anti (α)-PD-L1 or α-CD86 treatment during *in vitro* co-culture for Treg differentiation. **(B, C)** In blocking with α-PD-L1 or **(D, E)** α-CD86, **(B, D)** Foxp3^+^ cells percentage were quantified among CD4^+^ T cells after co-culture with specific APCs. **(C, E)** In co-culture with lean or obese VAT DCs, differentiated Foxp3^+^ cells were quantified. Data are means ± SEM, n= 2-5, *p<0.05, **p<0.01, ***p<0.001, ****p<0.0001 between groups as indicated. ## *p*<0.01 ND veh vs HFD veh. ND, normal diet; HFD, high-fat diet.

Given the distinct alterations in Foxp3 and phospho-AKT/-mTOR expressions observed in Tregs co-cultured with obese VAT DCs, we aimed to investigate the regulatory role of upstream signalling involving PD-L1 or CD86. These molecules were expressed at higher levels by obese VAT DCs, and we speculated that they might mediate Treg function. To explore this, anti (α)-PD-L1 or α-CD86 treatments were applied to the APCs in the *in vitro* co-culture for Treg differentiation (**Figure 6A**). In the lean state, inhibition of PD-L1 signalling resulted in lower Treg generation across all types of APCs (**Figure 6B**), underscoring the importance of PD-L1 molecules in inducing Foxp3 expression during Treg differentiation (25). However, when we blocked PD-L1 in obese VAT DCs, no change in the proportion of differentiated Tregs was observed (**Figure 6C**). Moreover, there were no changes of phospho-AKT expressions upon blocking of PD-L1 (**Supplementary Figures 5A, B**), suggesting that the regulation of decreased Treg conversion may be independent of PD-L1/AKT pathway. In contrast, inhibition of CD86 in the lean state demonstrated a nonspecific response, with all types of APCs (except SAT Mφs) exhibiting higher percentages of Foxp3^+^ Tregs (**Figure 6D**), but the total T cell numbers were decreased (**Supplementary Figure 5C**). Interestingly, obese VAT DCs also showed the induction of Foxp3^+^ Treg proportion at a similar level as lean VAT DCs (**Figure 6E**), yet the number of Foxp3^+^ Tregs were markedly decreased in the blockade of CD86 (**Supplementary Figure 5D**). Additionally, expression of phospho-AKT were non-specifically increased in co-culture with lean or obese VAT DCs, while other APCs showed decreased or no changes (**Supplementary Figures 5E, F**). These findings suggest that CD86/AKT pathway may not serve as the specific mechanism to regulate reduction of Treg differentiation by obese VAT DCs.

### 3.6 Obesity diminishes IL-33 production in DCs, which mediates the attenuation of Tregs induced by obese VAT DCs

**Figure 7:**
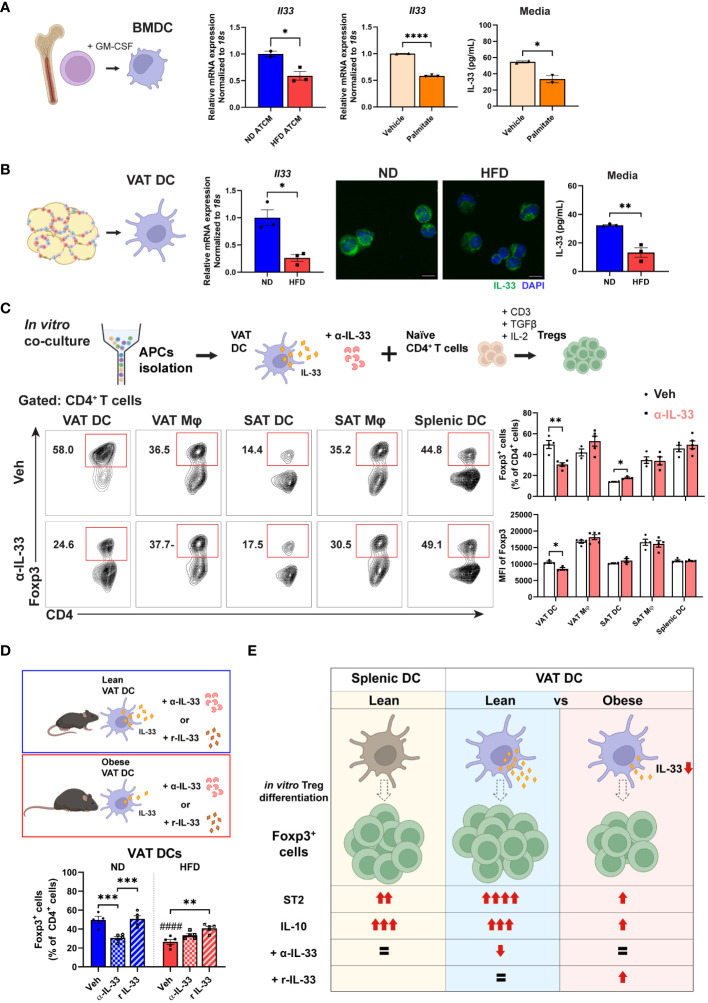
Impaired Treg differentiation via lower IL-33 production by obese VAT DC. **(A)** Mature BMDCs were treated with ND or HFD adipose tissue conditioned media (ATCM) for 24 h and mRNA expression of *Il33* was determined. Separately, BMDCs were treated with vehicle or palmitate-BSA for 24 h, and relative *Il33* mRNA expression and IL-33 secretion levels were determined. **(B)** VAT DCs were sorted from ND and HFD mice for determination of *Il33* mRNA expressions, IF staining of IL-33 and DAPI, and IL-33 secretion levels. **(C)** Schematic illustration and representative flow cytometry diagram of anti (α)-IL-33 treatment during *in vitro* co-culture for Treg differentiation. Foxp3^+^ cells and the MFI of Foxp3 were quantified after 5 days co-culture with specific type of APCs. **(D)** Schematic diagram and quantification of Foxp3^+^ cells after *in vitro* Treg differentiation with lean or obese VAT DCs under treatment with vehicle, α-IL-33, or recombinant IL-33 (r IL-33). **(E)** Proposed model of VAT DC as the critical regulator to direct *in vitro* Treg differentiation profile and function through IL-33 in obese conditions. Data are means ± SEM, n= 2-5, *p<0.05, **p<0.01, ***p<0.001, ****p<0.0001 between groups as indicated. ####*p*<0.0001 ND veh vs HFD veh. ND, normal diet; HFD, high-fat diet.

Given our findings demonstrating a reduction in ST2 expression both in VAT Tregs and in differentiated Tregs after co-culture with obese VAT DCs, we sought to investigate the potential involvement of IL-33 in the regulation of Tregs by DCs. Previous studies have indicated that bone marrow-derived dendritic cells (BMDCs) express IL-33 in the cytosol, which can be released in the basal state (41, 45). To explore whether obesity can alter IL-33 expression in DCs, we incubated BMDCs with AT-conditioned media (ATCM) from lean and obese VAT. Notably, a significant decrease in *Il33* mRNA expression was observed in BMDCs treated with HFD ATCM compared to ND ATCM. Additionally, palmitate treatment significantly reduced *Il33* mRNA expression as well as IL-33 cytokine secretion in BMDCs, indicating that high levels of saturated fat present in obese VAT can lead to reduced IL-33 expression by DCs (**Figure 7A**). Next, we aimed to evaluate the expression of IL-33 in VAT DCs. In sorted obese VAT DCs, a significantly lower *Il33* mRNA expression was confirmed compared to lean control. IF staining displayed high IL-33 expression in the cytosol of VAT DCs from lean mice, which was diminished in obese conditions. Additionally, obese VAT DCs exhibited decreased IL-33 cytokine secretion compared to lean ones (**Figure 7B**). Furthermore, we confirmed that other APCs showed no significant changes in *Il33* mRNA expressions in obesity (**Supplementary Figure 6A**). These results collectively suggest that obesity specifically decreases IL-33 expression in VAT DCs.

To ascertain whether IL-33 acts as the mediator in the reduction of Treg differentiation by obese VAT DCs, we treated sorted APCs from lean mice with α-IL33 in co-culture with naïve CD4^+^ T cells. Consistent with our findings, α-IL-33 treatment exclusively led to lower Treg differentiation as well as a reduced MFI of Foxp3 in co-culture with VAT DCs, but not with other APCs (**Figure 7C**). Furthermore, while α-IL-33 treatment did not alter Treg differentiation in co-culture with obese VAT DCs, treatment with rIL-33 enhanced Treg differentiation specifically in obese VAT DCs, but not in VAT macrophages (Mφs) (**Figure 7D**; **Supplementary Figure 6B**). Additionally, no alterations were detected in phospho-AKT expressions after α-IL-33 treatment (**Supplementary Figures 6C, D**), indicating that the action of IL-33 may be independent of AKT pathway. Altogether, these findings suggest specific action of IL-33 derived from VAT DCs as the key regulator of Treg differentiation, which is disrupted in obese situations.

In summary, our results demonstrate that obesity distinctively alters Treg and APC profiles in VAT, with different correlation profiles between DCs-Tregs and Mφs-Tregs. Furthermore, we identified VAT DCs as the primary APCs to drive Treg differentiation, particularly under obese conditions, showing decreased capacity and lower expression levels of ST2 and IL-10 in the differentiated Tregs. Obesity was found to lower IL-33 expression and secretion in VAT DCs, which was primarily responsible for the attenuated Treg generation. These results suggest a potential contribution of VAT DCs to the decreased Treg number in obese VAT (**Figure 7E**).

## 4 Discussion

A comprehensive understanding of AT Treg regulation during obesity is crucial for identifying strategies to modulate AT inflammation. The unique regulation of AT Tregs in distinct fat depots is recognized as a key factor contributing to the differential inflammatory status between obese VAT and SAT. This study reveals that obesity distinctly alters the profiles of both Tregs and APCs, highlighting obese VAT DC as the primary APC controlling Treg generation with distinctive phenotypes. Furthermore, our findings indicate that IL-33 derived from VAT DCs plays a critical role in mediating decreased Treg differentiation in obese situations, shedding light on depot-specific regulation of tissue inflammation and homeostasis in obesity. These results underscore the potential pivotal role of DCs in orchestrating the Treg population in obese VAT.

The interaction with resident APCs is a key mechanism governing the development and activation of Tregs within ATs (46). Previous *in vivo* studies have indicated the dependency of AT T cells on antigen presentation activity by APCs. Deletion of MHC-II molecules in APCs or ablation of CD11c^+^ cells demonstrated changes in VAT Tregs and Tconv profiles (19, 20, 22). Additionally, adipocyte-MHC-II expression has been reported to participate in the regulation of CD4^+^ T cells in obesity (47, 48). However, our studies emphasize APCs as the major MHC-II-expressing cells in VAT that professionally acquire, digest, and present antigens to T cells (22, 49). The unique TCR repertoire found in VAT Tregs implies the specificity of antigens presented by APCs, which can be regulated differently among various proficient APCs and influence local Treg accumulation (12, 20). This study demonstrates that different APCs derived from distinct fat depots have varying capabilities to induce Treg differentiation. Remarkably, VAT DC emerges as the most potent APC for producing Tregs in steady state, surpassing VAT Mφ, SAT DC, SAT Mφ, and splenic DC. These findings align with our prior studies identifying ATDC as the most professional APC for stimulating T cell proliferation, including Tregs (26, 42).

In situations of obesity, a specific reduction in Tregs was observed exclusively in VAT, accompanied by distinct correlation patterns between specific types of APCs and Tregs. Notably, our *in vitro* co-cultures demonstrate that VAT DCs, as opposed to VAT Mφs, exhibit diminished Treg differentiation capability in obese conditions, while SAT APCs and splenic DCs show no alterations. The increased numbers of both DCs and Mφs within visceral fat, recognized as a hallmark of obesity (22, 26), raise the question of whether these two types of APCs share a common role in orchestrating immune cell alterations within obese visceral fat. However, to date, the existence of various subtypes within specific types of APCs in obesity has made it challenging to delineate the differential capacities between ATDCs and ATMs through *in vivo* studies, given their overlapping definitive markers (22, 50, 51). In this study, we observed a rise in the frequency of antigen presentation molecules in tandem with the prevalence of APCs. However, the degrees of individual cellular expression of these molecules fluctuate between ATDCs and ATMs during obesity. This observation prompted us to re-evaluate their correlation and capacity to control Tregs using an *in vitro* system. Indeed, our *in vitro* co-culture system reveals distinct proficiency between DCs and Mφs in driving Treg generation in obese conditions, further supporting the notion of varied roles played by these APC subtypes in the context of obesity.

Among various DC subsets, a prior study has implicated plasmacytoid DCs (pDCs), expressing IFN-α, as contributors to reducing the Treg population in obese VAT (52). Despite their relatively minor quantity, pDCs play a more dominant role in antiviral responses, while conventional DCs (cDCs) excel in antigen presentation (27). In this study, we employ CD11c^+/hi^ to gate DCs, encompassing cDCs while excluding pDCs. By isolating cDCs, we can meticulously investigate their capacity to regulate Treg differentiation. Our findings reveal that obese VAT DCs consistently diminish Treg generation, both in an antigen non-specific and antigen-specific manner. Moreover, obese VAT DCs shape distinctive Treg features characterized by higher phospho-AKT/-mTOR, elevated PD-1, lower ST2, and reduced IL-10 expressions. These results suggest that VAT DCs themselves serve as a promising major mechanism for modulating Treg phenotype and features in obesity. The unique Treg profile may be attributed to different cellular and molecular mechanisms during interaction with ATDC, possibly involving the regulation of molecules associated with T cell interaction, such as antigen presentation molecules, and cytokine signaling, such as IL-33/ST2.

The relevance of IL-33/ST2 signaling as a regulator of Treg populations has been documented in both steady-state conditions and various diseases, including gastrointestinal disease, lung disease, cancer, multiple sclerosis, and other autoimmune models (53). High expression of ST2 has been identified as a unique marker for VAT Tregs compared to splenic Tregs, contributing to their modulation in obesity (21). In this study, we reinforce ST2 as the definitive marker for fat depot-specific Tregs, playing a role in obesity-specific regulation. Conversely, SAT Tregs exhibit low levels of ST2 expression and remain unaltered in obesity. TCR signaling activation induces transcription factors *Batf* and *Irf4*, targeting the expression of *Il1rl1* (ST2) (54). While ST2 expression in Tregs has been noted as a significant cell-intrinsic factor for VAT Treg accumulation, our study adds further evidence for the capacity of APCs to induce ST2 expression in Tregs. Notably, this process is exclusively inhibited in co-culture with obese VAT DCs. Thus, our findings suggest that the regulation of Tregs by VAT DCs in the context of obesity involves the modulation and action of ST2.

However, the reduction of Tregs in obese visceral fat through an ST2-dependent manner remains debated, as the loss of ST2 resulted in lower VAT Tregs in some studies (21), while others found no differences (32). Nonetheless, it is evident that IL-33 is crucial for VAT Treg survival and maintenance, as exogenous IL-33 induces their expansion both *in vivo* and *in vitro*, with or without the presence of ST2 (21, 32, 33). While mesenchymal-derived stromal cells have been reported as major sources of IL-33 in AT (34, 36, 37), IL-33 mRNA expression can also be found in epithelial cells, endothelial cells, adipocytes, and DCs (40, 53, 55). Recent research indicates that splenic DCs and BMDCs exceptionally express IL-33 in the cytosol, potentially releasing it through the perforin-2 channel, regulating ST2 expression on Tregs (41). Our findings reveal that VAT DCs express IL-33 in the cytosol, which can be secreted but is reduced in obese situations. Furthermore, our results demonstrate that the decreased IL-33 action by obese VAT DCs diminishes Treg differentiation, as blocking IL-33 in co-culture with lean VAT DCs specifically results in lower Treg conversion. Remarkably, exogenous IL-33 treatment can reverse the decreased Treg development by obese VAT DCs. Additionally, our findings suggest that obesity conditions, particularly a high concentration of saturated fat, lead to reduced IL-33 mRNA expression and cytokine release in DCs. This aligns with previous studies where high-lipid-droplets-DCs have lower capability to induce Treg differentiation than low-lipid-droplets-DCs (56). Thus, our data further emphasizes IL-33 derived from VAT DCs as the critical mechanism for modulating Treg populations in obesity. However, considering stromal cells to the IL-33 production, the impact of DC-derived IL-33 on the Treg function *in vivo* should be further investigated in future studies.

Costimulatory molecules, such as CD86 and PD-L1, have been identified as crucial regulators in Treg development (57–59). While the inhibitory effect of PD-L1 signaling on AKT/mTOR during Treg differentiation is known (59, 60), investigating PD-L1’s role in DC-Treg regulation *in vivo* poses challenges due to its expression by various cell types (61–64). In contrast, both B7 co-stimulatory molecules, CD80 and CD86, have been demonstrated as essential for Treg development in both *in vivo* and *in vitro* studies (23, 65). CD86 signaling induces phospho-AKT in T cells and is vital for Treg generation (58, 65–67). Our *in vitro* studies indicate that blocking PD-L1 hampers Treg differentiation by all APCs with unchanged phospho-AKT expressions. Conversely, blocking CD86 reduces T cell proliferation but increases Foxp3 proportions, suggesting a higher tendency to inhibit Tconv generation. Notably, blocking CD86 in obese DCs exhibits a similar impact on Treg generation as observed in lean DCs with higher phospho-AKT expressions. These results align with previous studies, yet discrepancy of Foxp3^+^ percentages were noted. These variations might be attributed to the presence of CD80 and various binding partners, such as CD28 and CTLA-4 (65, 66). Overall, these observations underscore the importance of PD-L1 and CD86 in Treg development. Furthermore, additional regulatory factors may exist and influence AKT pathway, as they alone may not suffice to explain the obese depot-specific regulation of Tregs. Further investigations are warranted to comprehensively understand the specific role and regulatory mechanism of antigen-presenting molecules in APCs during obesity.

In summary, this study underscores the significance of VAT DCs as major regulators in driving and shaping Treg population in obese conditions. Intriguingly, our findings reveal that obesity, particularly with a high saturated fat content, downregulates IL-33 expression and secretion in VAT DCs, thereby mediating decreased Treg differentiation. These insights potentially elucidate the contribution of VAT DCs to the inflammatory milieu in obese visceral fat. Moreover, our study enhances understanding of leukocyte organization in distinct fat depots during obesity. Further investigation into the *in vivo* regulation of Tregs by VAT DCs, particularly in the context of their contribution to the overall Treg population during obesity, remains warranted. Additionally, delving into the detailed mechanisms through which obesity specifically alters these attributes and functions in VAT DCs holds promise for identifying future therapeutic targets to mitigate Treg reduction in obesity.

## Data availability statement

The original contributions presented in the study are included in the article/**Supplementary Material**. Further inquiries can be directed to the corresponding authors.

## Ethics statement

The animal study was approved by Institutional Animal Care and Use Committee at the Soonchunhyang University. The study was conducted in accordance with the local legislation and institutional requirements.

## Author contributions

SSo: Conceptualization, Data curation, Formal analysis, Investigation, Methodology, Validation, Visualization, Writing – original draft, Writing – review & editing. SSh: Investigation, Methodology, Writing – review & editing. DN: Investigation, Methodology, Writing – review & editing. MA: Investigation, Methodology, Writing – review & editing. ER: Investigation, Methodology, Writing – review & editing. YL: Conceptualization, Supervision, Writing – review & editing, Validation. KC: Conceptualization, Supervision, Writing – review & editing, Validation.
