# Supplementary material for: Obese visceral adipose dendritic cells downregulate regulatory T cell development through IL-33
Source: Front Immunol. 2024 Mar 19;15:1335651. doi: 10.3389/fimmu.2024.1335651 (PMC10985834; doi:10.3389/fimmu.2024.1335651)
Supplement: Supplementary file 1 [file DataSheet_1.docx]

Supplementary Material

# Supplementary Data

## Supplementary Figures


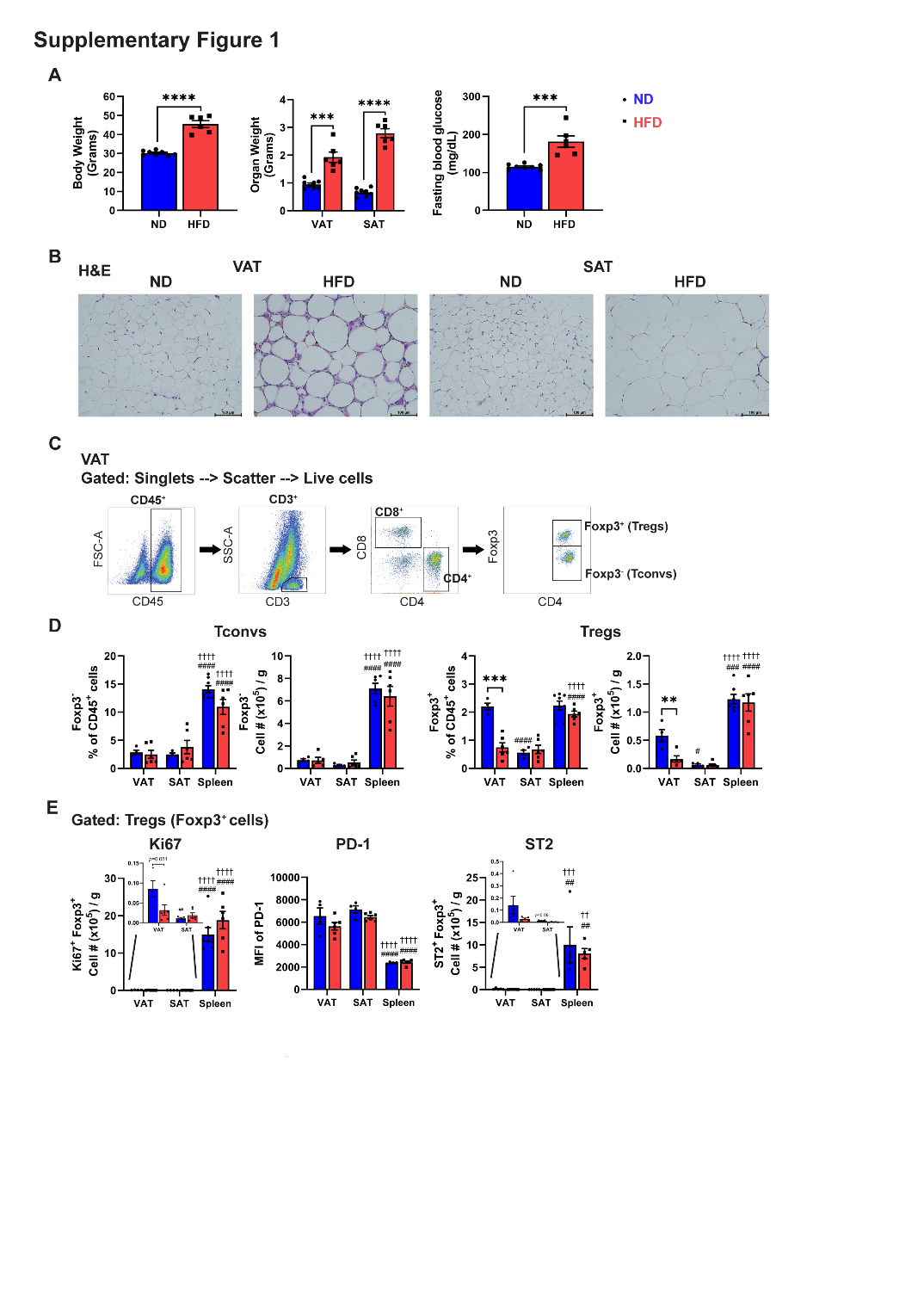


Supplementary Figure 1. Depot-specific reduction of regulatory T cell (Treg) number in obese VAT.

C57BL/6 mice were induced to diet-induced obesity (DIO) by 16 weeks of high-fat diet (HFD). At the end of DIO, **(A)** final body weight, organ weight and fasting blood glucose levels were measured. **(B)** Visceral adipose tissue (VAT) and subcutaneous AT (SAT) were sectioned and stained with hematoxylin and eosin (H&E) staining. Representative pictures were taken with 20X magnifications. **(C)** Gating strategy for flow cytometry analysis of T cells. Live CD45^+^ cells were gated for CD3^+^ and followed by CD4^+^ then Foxp3^+^ (Tregs) or Foxp3^-^ (Tconvs). **(D)** Quantification of Tconvs and Tregs in percentage of leukocytes and total cell number per gram. **(E)** Quantitation of cell number of Ki67^+^ Tregs, MFI of PD-1, and cell number of ST2^+^ Tregs. Data are means ± SEM, n= 4-6, ** *p*<0.01, *** *p*<0.001, **** *p*<0.0001 ND vs HFD. ## *p*<0.01, #### *p*<0.0001 VAT vs SAT or spleen. †† *p*<0.01, ††† *p*<0.001, †††† *p*<0.0001 SAT vs spleen. ND=normal diet, HFD= high-fat diet.


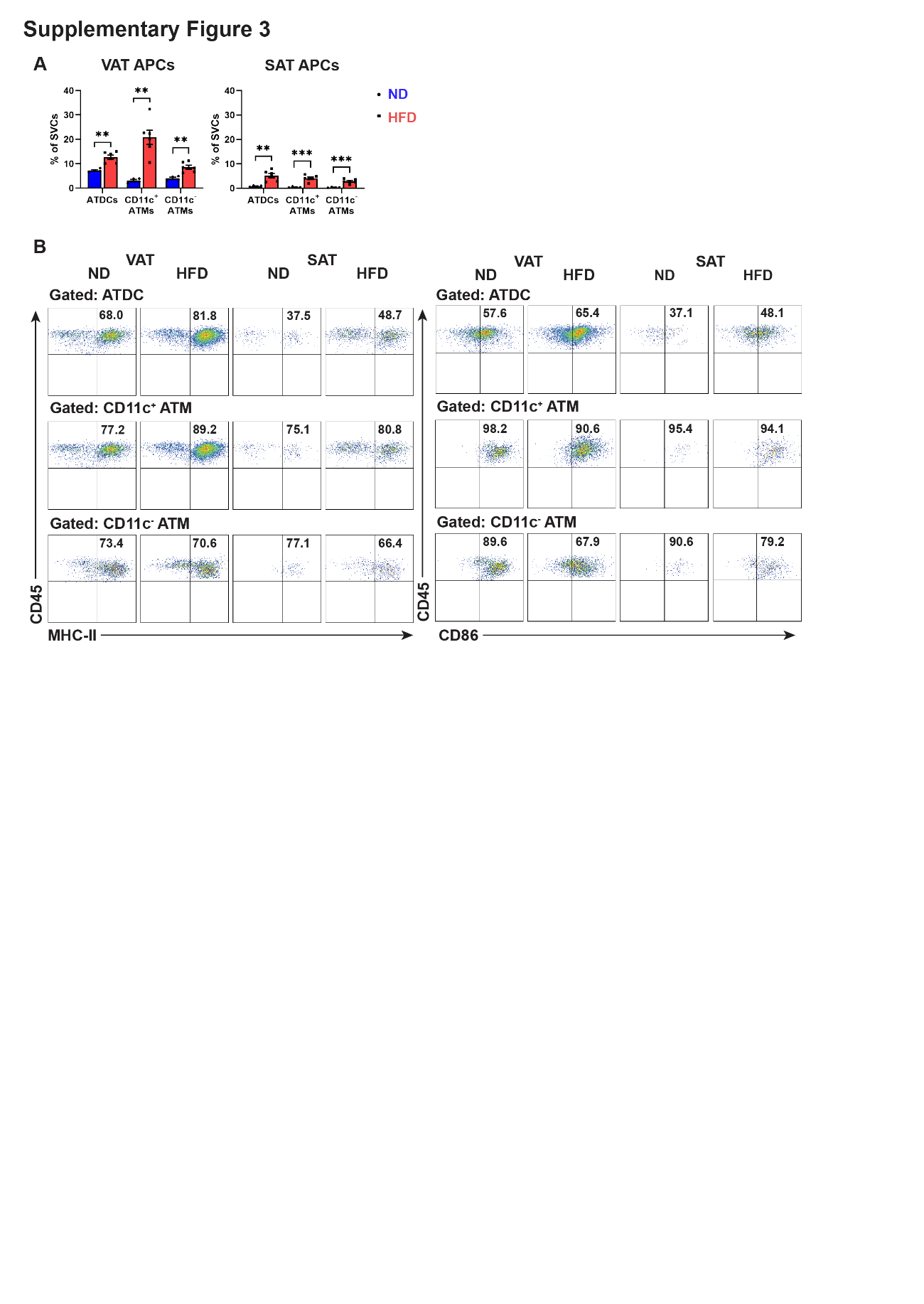


Supplementary Figure 2. Differential regulation of antigen presenting cells (APCs) accumulation and maturation in obese VAT and SAT.

**(A)** Quantification of ATDC (CD64^-^ CD11c^+^), CD11c^+^ ATM (CD64^+^ CD11c^+^), and CD11c^-^ ATM (CD64^+^ CD11c^-^) in percentage of SVCs. **(B)** Representative flow cytometry diagram of MHC-II^+^ and CD86^+^ expression in ATDC, CD11c^+^ ATM, and CD11c^-^ ATM in VAT and SAT. Data are means ± SEM, n= 4-6, ** *p*<0.01, *** *p*<0.001 ND vs HFD. ND=normal diet, HFD= high-fat diet.


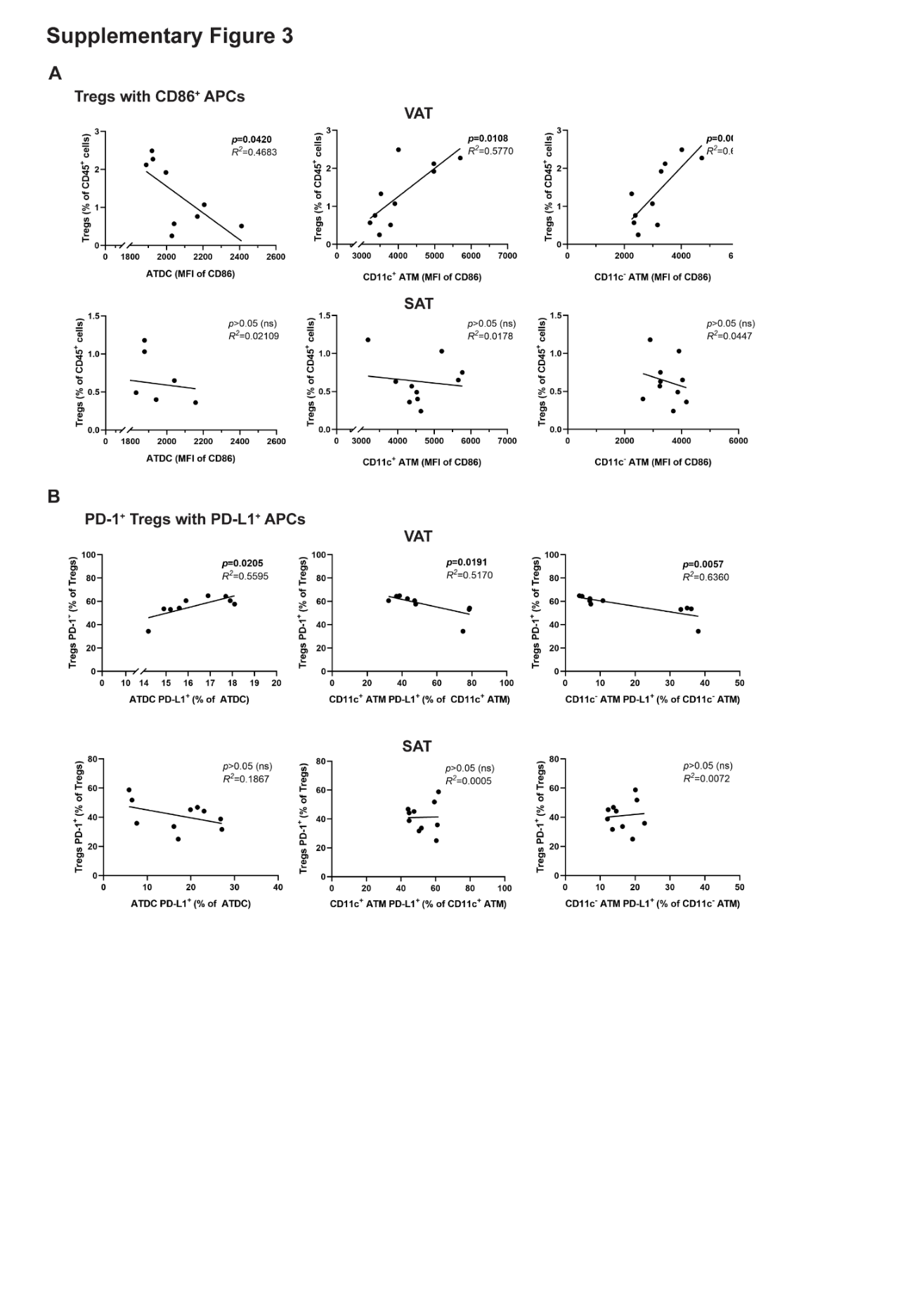


Supplementary Figure 3. Distinctive correlation patterns between Tregs and APCs in obese VAT and SAT.

**(A)** Correlation analysis of MFI of CD86 in APCs with Tregs in percentage of leukocytes. **(B)** Correlation analysis of PD-L1^+^ APCs with PD-1^+^ Tregs. Data are means ± SEM, n= 4-6, ND=normal diet, HFD= high-fat diet; ns= not significant.


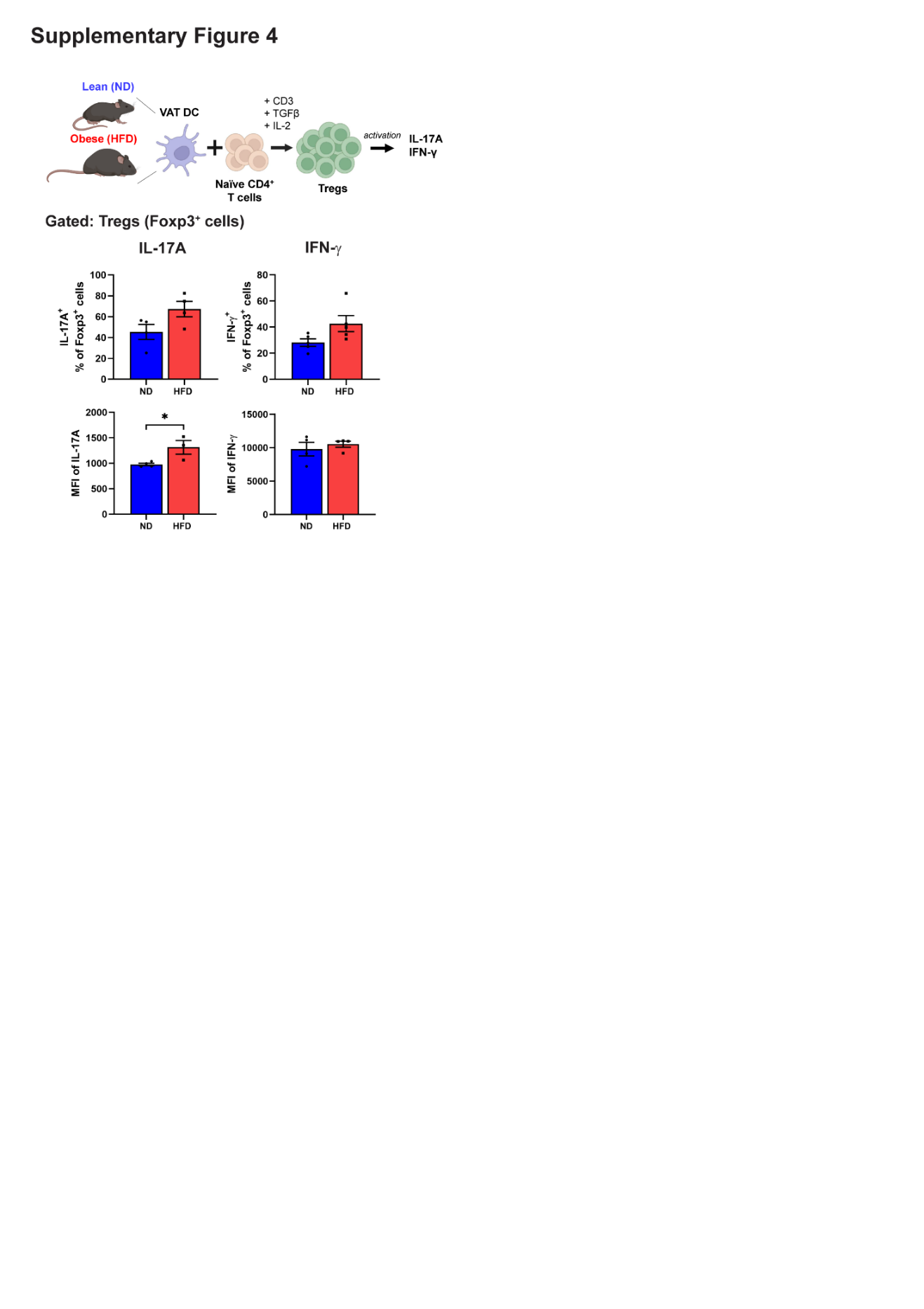


Supplementary Figure 4. Expressions of IL-17A and IFN-γ in Tregs co-cultured with VAT DCs.

After cells activation and permeabilization, intracellular cytokine expressions of IL-17A and IFN-γ were quantified from *in vitro*-differentiated Foxp3^+^ cells in co-culture with VAT DCs. Data are means ± SEM, n= 3-5, * *p*<0.05 ND vs HFD. ND=normal diet, HFD= high-fat diet.


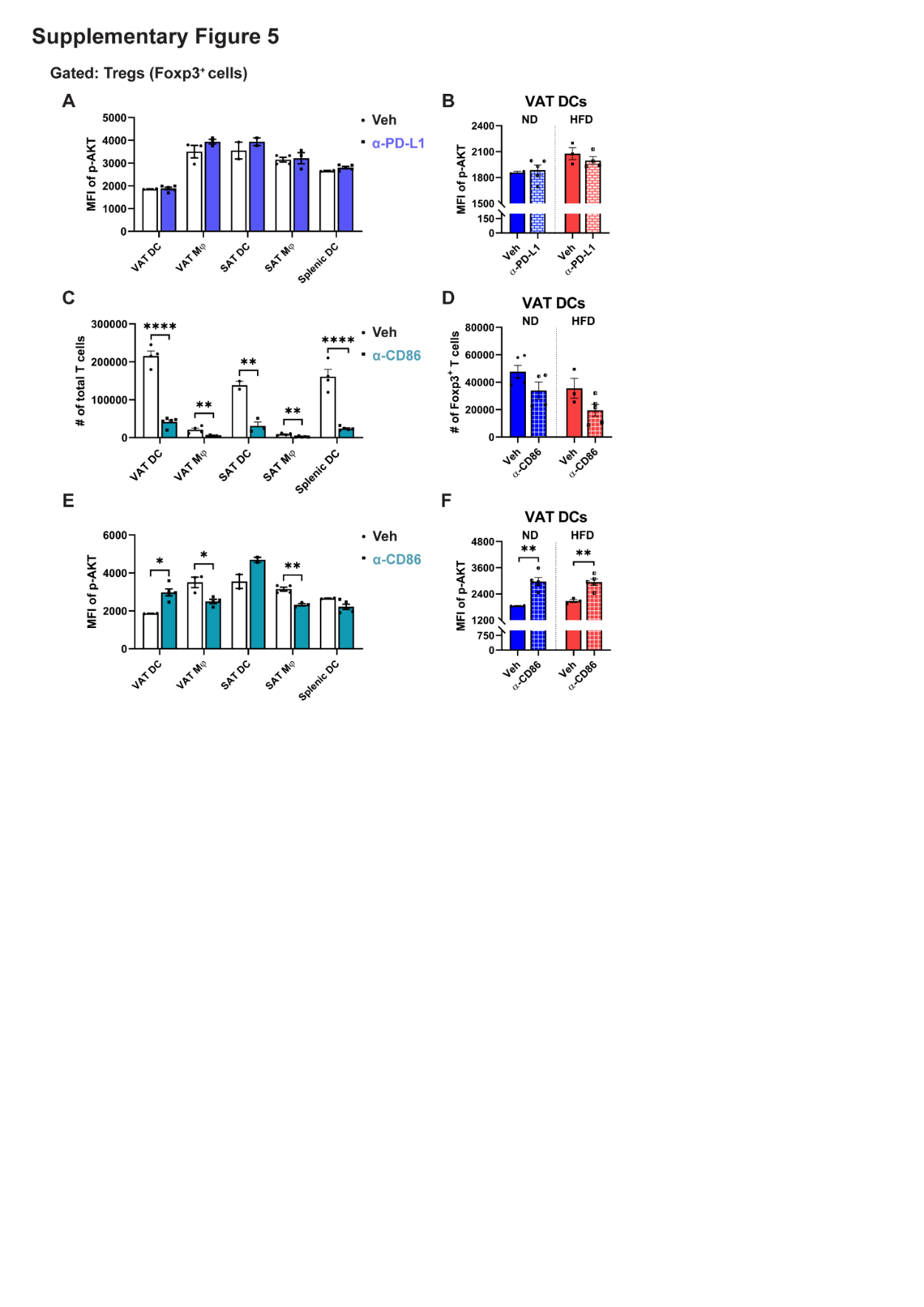


Supplementary Figure 5. Profiles of *in vitro* differentiated Tregs following co-culture with APCs with PD-L1 or CD86 blockade.

(A) In blocking with vehicle or α-PD-L1, MFI of phospho-AKT (Ser473) were quantified in Tregs after co-culture with specific APCs. (B) MFI of p-AKT in Tregs co-culture with lean or obese VAT DCs. (C-F) In blocking with vehicle or α-CD86, (C) total absolute number of CD4^+^ T cells were determined after co-culture with specific APCs in lean condition with flow cytometry. (D) Absolute number of Foxp3^+^ Tregs after co-culture with lean or obese VAT DCs. (E) MFI of p-AKT in Tregs after co-culture with indicated APCs. (F) MFI of p-AKT in Tregs co-cultured with lean or obese VAT DCs. Data are means ± SEM, n= 2-5, * *p*<0.05, ** *p*<0.01, **** *p*<0.0001 ND vs HFD. ND=normal diet, HFD= high-fat diet.


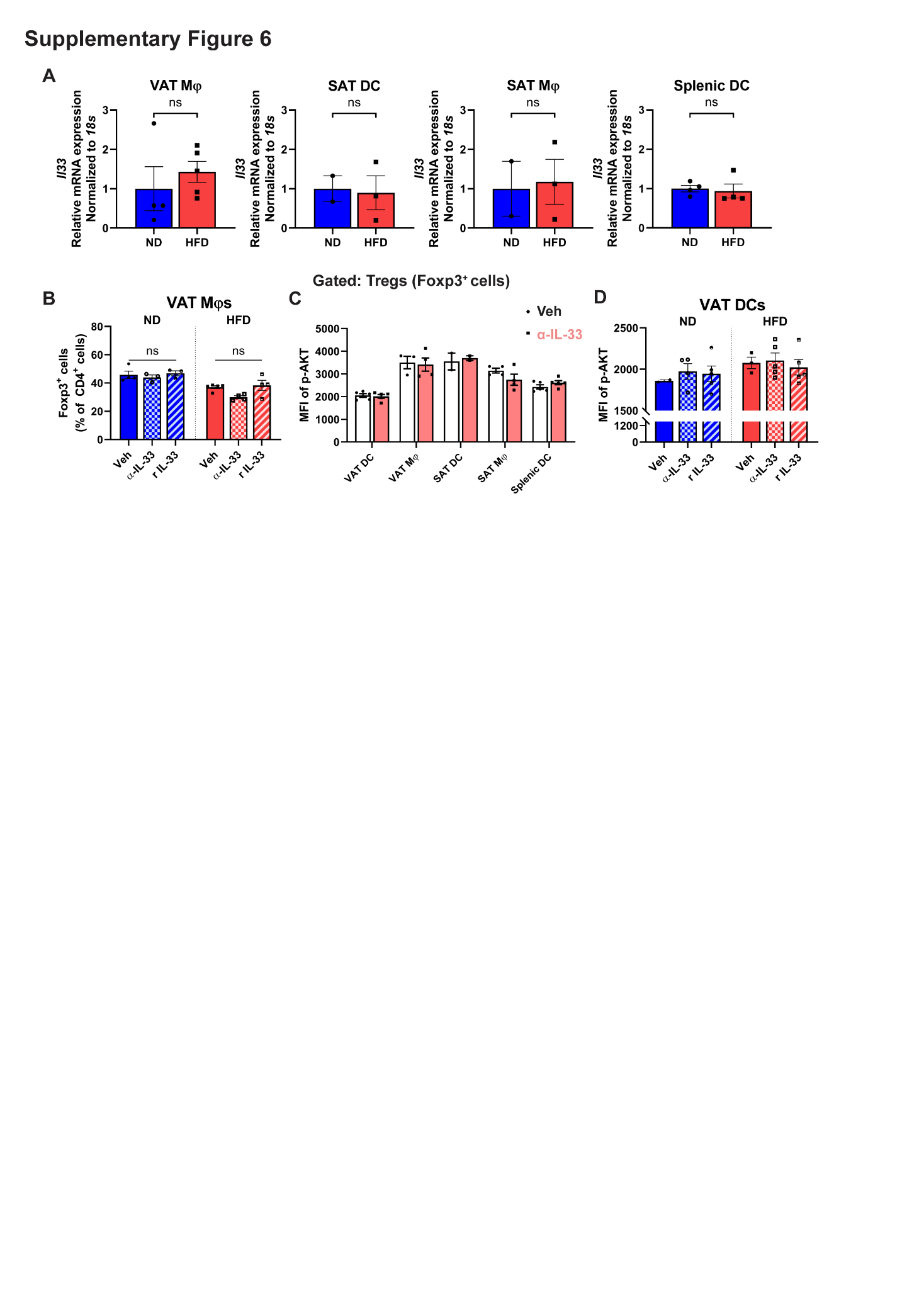


Supplementary Figure 6. Effects of obesity on the IL-33 production of adipose immune cells.

**(A)** Relative mRNA expressions of *Il33* in sorted cells VAT Mφs, SAT DCs, SAT Mφs, and splenic DCs from lean and obese mice. **(B)** Quantification of Foxp3^+^ cells after *in vitro* Treg differentiation with lean or obese VAT Mφs under treatment with vehicle, α-IL-33, or recombinant IL-33 (rIL-33). **(C)** MFI of phospho-AKT (Ser473) in the *in vitro* differentiated Tregs co-cultured with specific APCs in the presence of vehicle or α-IL-33. **(D)** MFI of phospho-AKT (Ser473) in the *in vitro* differentiated Tregs after co-culture with lean or obese VAT DCs under treatment with vehicle, α-IL-33, or recombinant IL-33 (r IL-33). Data are means ± SEM, n= 2-5, ns= non-significant. ND=normal diet, HFD= high-fat diet.

## Supplementary Table

**Supplementary Table 1. List of antibodies used in flow cytometry and immunostaining.**

| **Flow cytometry** | | | | |
| --- | --- | --- | --- | --- |
| **Antibody** | **Clone** | **Fluorochrome** | **Identifier** | **Sources** |
| Fixable Viability Dye |  | eFluor® 450 | 65-0863-14 | eBioscience™ |
| Fixable Viability Dye |  | eFluor® 506 | 65-0866-14 | eBioscience™ |
| CD45 Monoclonal Antibody | 30-F11 | eFluor® 450 | 48-0451-82 | eBioscience™ |
| CD45 Monoclonal Antibody | 30-F11 | APC-eFluor® 780 | 47-0451-82 | eBioscience™ |
| Mouse anti-Mouse CD64 a and b Alloantigen | X54-5/7.1 | PE | BD558455 | BD Pharmingen™ |
| CD11c Monoclonal Antibody | N418 | APC | 17-0114-82 | eBioscience™ |
| MHC Class II (I-A/I-E) Monoclonal Antibody | M5/114.15.2 | APC-eFluor® 780 | 47-5321-82 | eBioscience™ |
| CD86 (B7-2) Monoclonal Antibody | GL1 | APC | 17-0862-82 | eBioscience™ |
| CD274 (PD-L1, B7-H1) Monoclonal Antibody | MIH5 | PE-Cyanine7 | 25-5982-82 | eBioscience™ |
| CD3e Monoclonal Antibody | 145-2C11 | PerCP-Cyanine5.5 | 45-0031-82 | eBioscience™ |
| CD4 Monoclonal Antibody | RM4-5 | eFluor® 450 | 48-0042-82 | eBioscience™ |
| CD8a Monoclonal Antibody | 53-6.7 | PE-Cyanine7 | 25-0081-82 | eBioscience™ |
| Foxp3 Monoclonal Antibody | FJK-16S | PE | 12-5773-82 | eBioscience™ |
| Ki-67 Monoclonal Antibody | SolA15 | APC | 14-5698-82 | eBioscience™ |
| CD279 (PD-1) Monoclonal Antibody | J43 | APC | 17-9985-82 | eBioscience™ |
| IL-33R (ST2) Monoclonal Antibody | RMST2-2 | APC | 47-9335-82 | eBioscience™ |
| Phospho-Akt (Ser473) XP® Rabbit mAb | D9E |  | 4060S | CST |
| Phospho-mTOR (Ser2448) Rabbit mAb | 49F9 |  | 2976S | CST |
| IL-10 Monoclonal Antibody | JES5-16E3 | APC | 17-7101-82 | eBioscience™ |
| IL-17A Monoclonal Antibody | eBio17B7 | FITC | 11-7177-81 | eBioscience™ |
| IFN gamma Monoclonal Antibody | XMG1.2 | APC | 17-7311-82 | eBioscience™ |
| CD16/CD32 Monoclonal Antibody | 93 |  | 14-0161-82 | eBioscience™ |
| CD3e Monoclonal Antibody | 145-2C11 | PE | 12-0031-82 | eBioscience™ |
| Goat anti-Rabbit IgG (H+L) |  | Alexa Fluor® 488 | A11034 | Invitrogen |
| **Immunostaining** | | | | |
| **Antibody** | **Clone** | **Fluorochrome** | **Identifier** | **Sources** |
| CD4 Monoclonal Antibody | RM4-5 | FITC | 11-0042-81 | eBioscience™ |
| Caveolin-1 Monoclonal Antibody | 7C8 |  | sc-53564 | Santa Cruz |
| Mouse IL-33 Polyclonal Antibody |  |  | AF3626 | R&D Systems |
| Goat anti-Mouse IgG (H+L) |  | Alexa Fluor® 488 | A11001 | Invitrogen |
| Donkey anti-goat IgG (H+L) |  | Alexa Fluor® 488 | A11055 | Invitrogen |
